# Short-Term Treatment with the Urease Inhibitor N-(n-Butyl) Thiophosphoric Triamide (NBPT) Alters Urea Assimilation and Modulates Transcriptional Profiles of Genes Involved in Primary and Secondary Metabolism in Maize Seedlings

**DOI:** 10.3389/fpls.2016.00845

**Published:** 2016-06-22

**Authors:** Laura Zanin, Silvia Venuti, Nicola Tomasi, Anita Zamboni, Rita M. De Brito Francisco, Zeno Varanini, Roberto Pinton

**Affiliations:** ^1^Dipartimento di Scienze Agroalimentari, Ambientali e Animali, University of UdineUdine, Italy; ^2^Department of Biotechnology, University of VeronaVerona, Italy; ^3^Institute of Plant Biology, University of ZurichZurich, Switzerland

**Keywords:** gene expression, microarray, nitrogen fertilizers, nitrogen nutrition, root uptake, urea, urea metabolism, *Zea mays*

## Abstract

To limit nitrogen (N) losses from the soil, it has been suggested to provide urea to crops in conjunction with the urease inhibitor N-(n-butyl) thiophosphoric triamide (NBPT). However, recent studies reported that NBPT affects urea uptake and urease activity in plants. To shed light on these latter aspects, the effects of NBPT were studied analysing transcriptomic and metabolic changes occurring in urea-fed maize seedlings after a short-term exposure to the inhibitor. We provide evidence that NBPT treatment led to a wide reprogramming of plant metabolism. NBPT inhibited the activity of endogenous urease limiting the release and assimilation of ureic-ammonium, with a simultaneous accumulation of urea in plant tissues. Furthermore, NBPT determined changes in the glutamine, glutamate, and asparagine contents. Microarray data indicate that NBPT affects ureic-N assimilation and primary metabolism, such as glycolysis, TCA cycle, and electron transport chain, while activates the phenylalanine/tyrosine-derivative pathway. Moreover, the expression of genes relating to the transport and complexation of divalent metals was strongly modulated by NBPT. Data here presented suggest that when NBPT is provided in conjunction with urea an imbalance between C and N compounds might occur in plant cells. Under this condition, root cells also seem to activate a response to maintain the homeostasis of some micronutrients.

## Introduction

It has been estimated that the global consumption of maize will double before 2050, with this cereal becoming the crop with the greatest production worldwide (Rosegrant et al., 2008).

Due to the low nitrogen (N) use efficiency (< 33%; Raun and Johnson, 1999), large amounts of N fertilizer have to be applied to sustain a high productivity of cultivated maize.

Urea represents the major form of N fertilizer applied worldwide (>50%; Heffer and Prud'hommer, 2014). During the last years a large number of studies demonstrated that roots of higher plants, including crops, possess dedicated transport systems for the acquisition of external urea. When present at low concentration in the soil, urea is taken up by plants through DUR3, a specific transporter present in the plasma membrane of root cells. The DUR3 transporter belongs to the sodium:solute symporter (SSS) family and is the major component of the high affinity urea transport system in plants (Kojima et al., 2007). Several aquaporins have been described as components of a low affinity acquisition system, although their role in urea nutrition remains to be clarified (Gu et al., 2012; Yang et al., 2015; for review see Kojima et al., 2006).

It has been suggested that more than the assimilation, the limiting factor for an efficient use of urea in crop plants is the slow uptake (Wang et al., 2013), which in turn depends on urea persistence in the soil. Once in the soil the stability of urea is affected by the presence of urease, an ubiquitous enzyme released by the microbial population or deriving from the decomposition of organic matter. This enzyme catalyzes the rapid hydrolysis of urea releasing carbon dioxide and ammonia, a N form which might be lost from the soil through volatilization (Dawar et al., 2011; Soares et al., 2012).

In order to delay urea hydrolysis, it has been proposed to apply urea in association with urease inhibitors (Zaman et al., 2008). In co-formulation with urease inhibitors, urea is available as an intact molecule for plant acquisition during a long term, acting essentially as a controlled (or slow) release N-fertilizer (Trenkel, 2010).

Several chemical compounds have been described to act as urease inhibitors (Kiss and Simihaian, 2002). Today the most promising and tested urease inhibitor applied in soils is N-(n-butyl) thiophosphoric triamide (NBPT), often formulated into granular urea fertilizers. This compound is a structural analog of urea (Medina and Radel, 1988) and acts with a mixed inhibition on urease activity (Juan et al., 2009). NBPT forms stable complexes with urease (Watson, 2005) coordinating both nickel (Ni) atoms of the urease active site and binding the oxygen atom of the urea-derived carbamate (Manunza et al., 1999).

Although NBPT is widely used, very few information is available on its interaction with the environment, the effects on soil and water quality, on animal health, and also on plant growth (for review see National Industrial Chemicals Notification and Assessment Scheme, 1997; Arora and Srivastava, 2013). Some studies showed that NBPT can affect soil characteristics, such as pH, temperature, and moisture content (Hendrickson and O'Connor, 1987; Hendrickson and Douglass, 1993; Sigunga et al., 2002; Clough et al., 2004), and lead to phytotoxic symptoms (Krogmeier et al., 1989; Bremner, 1995; Watson, 2000).

In a previous study (Zanin et al., 2015a), it was demonstrated that the presence of NBPT in the nutrient solution limited the capacity of plants to use urea as a N-source, resulting in a limited net-uptake rate of urea and in a reduced accumulation of ureic-N in maize seedlings. At least in part, this effect might be the consequence of NBPT uptake by roots. Indeed, in pea and spinach, NBPT was detected in plant tissues when roots were treated with a high concentration of this molecule (Cruchaga et al., 2011). This evidence highlights that NBPT can cross the root plasma membrane (Cruchaga et al., 2011) although the transport mechanism remains to be defined. Plant metabolism was affected by the accumulation of the urease inhibitor within the cells, as shown by a lower endogenous urease activity. This effect in turn determined an increase of the urea internal pool and a strong reduction of the ammonium levels in plant tissues (Artola et al., 2011; Cruchaga et al., 2011; Zanin et al., 2015a). Also the downstream reactions of the cytosolic urea assimilation were affected by NBPT, as indicated by the low glutamine synthetase activity (Artola et al., 2011) and the low expression of genes coding for glutamine and asparagine synthetases (Zanin et al., 2015a). Indeed, these enzymes might play a key role in the assimilation of ammonium released by the cytosolic hydrolysis of urea (as proposed by Mérigout et al., 2008; for review see Pinton et al., 2016).

Often the treatment with the combination of urea and NBPT is associated with leaf tip scorch and necrotic margins of laminar leaves (Krogmeier et al., 1989; Watson and Miller, 1996; Artola et al., 2011; Cruchaga et al., 2011). Although it is unclear if these visible symptoms are indirectly due to a cytotoxic effect of over-accumulated urea, the inhibitor is known to alter the first steps of ureic-N assimilation (Cruchaga et al., 2011; Zanin et al., 2015a). No information is available on possible further influences of NBPT on primary and secondary plant metabolism.

In the present work, maize seedlings were used for a transcriptomic analysis aiming to provide an overview of metabolic changes occurring in plants when NBPT is applied. Changes in the amino acid profiles as well in the urease activity were also monitored.

## Materials and methods

### Plant material and growth conditions

Maize seeds (*Zea mays* L., inbred line PR33T56, Pioneer Hybrid Italia S.p.A.) were germinated over aerated 0.5 mM CaSO_4_ solution in a dark growth chamber at 25°C. After 3 days, the seedlings were transferred into an aerated hydroponic system in a controlled climatic conditions: day/night photoperiod, 16/8 h; light intensity, 220 μmol m^−2^ s^−1^; temperature (day/night) 25/20°C; relative humidity 70 to 80%. After 2 days (5 day old) plants were transferred to a N-free nutrient solution (*Control* treatment) containing (μM): KCl 5; CaSO_4_ 500; MgSO_4_ 100; KH_2_PO_4_ 175; NaFe-EDTA 20; H_3_BO_3_ 2.5; MnSO_4_ 0.2; ZnSO_4_ 0.2; CuSO_4_ 0.05; Na_2_MoO_4_ 0.05. When present nitrogen was added to nutrient solution in form of urea [0.5 mM CO(NH_2_)_2_] (*Urea* treatment). The urease inhibitor NBPT [N(n-butyl) thiophosphoric triamide, Apollo Scientific Ltd] was applied in conjunction with urea (*Urea*+*NBPT* treatment) at a concentration of 0.897 μM, corresponding to 0.5% w/w of urea, that is the amount commonly used in the commercial formulation of NBPT-urea fertilizer. The pH of solution was adjusted to pH 6.0 with potassium hydroxide (KOH).

Physiological and transcriptional analyses were performed on 5-day-old maize plants exposed up to 24 h to the different N treatments. Nitrogen sources were supplied to nutrient solution after 1 h from the beginning of the light phase (*T*_0_ = 0 h of treatment). After 8 and 24 h of treatment, pool of six plants for each sample were analyzed immediately for physiological experiments or stored at −80°C until further processing for molecular works.

### Molecular work: microarray analyses and real-time RT-PCR validation

RNA extractions were performed using the Invisorb Spin Plant RNA kit (Stratec Molecular) as reported in the manufacturer's instructions. Maize roots (70 mg) were homogenized in liquid nitrogen and the powder was mixed with 900 μl of DCT solution and dithiothreitol according to the suppliers' instructions. The RNA was evaluated in an agarose/ formaldehyde gel and quantified using a spectrophotometer Nanodrop 2000 instrument (Thermo Scientific). For the microarray analysis, the RNA quality and quantity were determined using a Bioanalyzer Chip RNA 6000 series II (Agilent) and three independent biological replicates were used, for a total of 6 hybridizations. The cDNA synthesis, labeling, hybridization and washing reactions were performed according to the NimbleGen Arrays User's Guide (www.nimblegen.com). Each hybridization was carried out on a NimbleGen microarray (maize chip 12 × 135K Arrays from Roche), representing 59756 transcripts predicted from the B73 reference genome (ftp.maizesequence.org/current/filtered-set/ZmB73_5b_FGS_cdna.fasta.gz). A complete description of the chip is available at the Gene Expression Omnibus (www.ncbi.nlm.nih.gov/geo) under the series entry (GPL17540). The microarray was scanned using an Axon GenePix 4400 (Molecular Devices) at 532 nm (Cy-3 absorption peak) and GenePix Pro7 software (Molecular Devices) according to the manufacturers' instructions. Images were analyzed using NimbleScan v2.5 software (Roche), which produces Pair Files containing the raw signal intensity data for each probe and Calls Files with normalized expression data- (quantile normalization) derived probe summarization through RMA analysis (Irizarry et al., 2003). Analysis of normalized data (Calls Files) was performed using the open source software of the Bioconductor project (Gentleman et al., 2004) with the statistical R programming language (Ihaka and Gentleman, 1996). Differentially expressed probes were identified by linear model analysis (Smyth, 2005) using the LIMMA package and applying Bayesian correction, adjusted *P* ≤ 0.05, *n* = 3, *FC* ≥ |2.00|. All microarray expression data are available at the Gene Expression Omnibus (www.ncbi.nlm.nih.gov/geo) under the series entry GSE76828 and GSE53102.

Gene ontology (GO) analysis and GO enrichment was performed using the Singular Enrichment Analysis (SEA) of AgriGO (Du et al., 2010) with *Zea mays* 6a (http://genome.jgi.doe.gov/; Schnable et al., 2009) as custom reference background. Hypergeometric tests with Yekutieli as multi-test adjusted method were performed using the default parameters to adjust the *P*-value.

Visualization of transcript expression differences within specific pathways was carried out using *MapMan v.3.5.1R2* (Usadel et al., 2009) with the mapping file provided by the *MapMan* homepage (*Zmays_181;* http://mapman.gabipd.org/) and custom files for the pathways: urea assimilation and primary metabolism (Supplementary Table 1, Supplementary Figure 1) and phenylpropanoid pathway (Supplementary Table 2, Supplementary Figure 2).

To validate the microarray results, real-time RT–PCR analyses were performed. Total RNA was treated with 1 U mg^−1^ RNA of DNase I (Sigma Aldrich) and cDNA was synthesized from 1 μg of RNA following the application protocol of the manufacturers [42°C for 1 h with 1 pmol of oligo d(T)_23_VN (Sigma Aldrich); 15 U of Prime RNase Inhibitor (Eppendorf); 10 U of M-MulV RNase H^−^ (Finnzymes)]. After RNA digestion with 1 U of RNase A (USB) for 1 h at 37°C, gene expression analyses were performed by adding 0.16 μl of the cDNA to the real-time RT-PCR complete mix, FluoCycle™ sybr green (20 μl final volume; Euroclone, Pero, Italy), in a DNA Engine Opticon Real Time PCR Detection system (Biorad). Real-time RT-PCRs for 15 genes identified in the microarray comparisons were performed (Supplementary Table 3). The primers were designed using Primer3 software (Koressaar and Remm, 2007; Untergasser et al., 2012) and they were synthesized by Sigma Aldrich (Supplementary Table 3). The analyses of real-time results were performed using Opticon Monitor 2 software (Biorad) and the qPCR package (version 1.1–8; www.dr-spiess.de/qpcR.html) for the statistical R software (version 2.9.0). Efficiencies of amplification were calculated following the authors' indications (Ritz and Spiess, 2008). Real-time RT-PCR results were validated using two reference genes, on *ZmGPDH* and *ZmTUA*. Data were normalized with respect to the transcript level of the reference genes using the 2^−ΔΔCT^ method, where ΔΔCT = (C_T, Target_ − C_T, HK_)_Time x_ − (C_T, Target_ − C_T, HK_)_Time 0_ (Livak and Schmittgen, 2001).

### Measurement of nitrogen, urea, and ammonium content in maize tissues

After 24 h of treatment, samples of shoots and roots of maize were dried and their total N content was determined using a Carlo-Erba CHN analyser. For urea and ammonium determination, leaves and roots of maize were sampled and processed as described by Witte et al. (2002). The urea content was quantified using the diacetyl monoxime and thiosemicarbazide reagents and measuring the absorbance at 527 nm. The ammonium quantification was performed using the Barthelot reagent (EN ISO 11732) on a San^++^ Autoanalyzer (Skalar), the absorbance was determined at 660 nm.

### Analyses of amino acid profiles

Plant samples (150 mg) were ground in liquid N_2_ and homogenized in 600 μl of water:chloroform:methanol (3:5:12 v/v; Hacham et al., 2002). The samples were centrifuged at 18000 *g* for 5 min. The supernatant was collected and 750 μl water:chloroform (4.5:3) was added and the samples were centrifuged at 18000 *g* for 5 min. The supernatants were pooled into a fresh tube, completely dried at the speed vacuum and solubilized in 100 μl of water. The samples were transferred to liquid chromatography (LC) vials and analyzed by ultraperformance LC-mass spectrometry (UPLC-MS). Amino acids analysis was performed using an UPLC (Thermo Scientific Dionex UltiMate 3000) coupled to a Bruker Compact Electrospray Ionization-Quadrupole-Time-of-Flight (ESI-Q-TOF; Bruker Daltonics). Liquid chromatography separation was performed on a BEH Amide column (1.7 μm, 2.1 × 150 mm, Waters) as described (Guo et al., 2013). The mobile phase was composed of solvent A (water, 10 mM ammonium formate, 0.15% formic acid) and solvent B (acetonitrile, 2 mM ammonium formate, 0.15% formic acid) with a gradient elution: 0–6 min, 15–20% A; 6−10 min, 20–30% A; 10–12 min, 30–40% A; 12–18 min—equilibration to initial conditions. The flow rate was set up to 0.3 ml min^−1^ and 5 μl of each sample was injected. Electrospray ionization source was operated in positive mode and parameters were set as follow: gas temperature, 220°C; drying gas, 9 l min^−1^; nebulizer, 2.2 Bar; capillary voltage, 4500 V; end plate offset, 500 V. The instrument was set to acquire m/z 50–1300. The solvents used were LC-MS/MS grade (Chemie Brunschwig) and all amino acids were purchased from Sigma Aldrich (Switzerland).

All data were analyzed using Data Analysis (version 4.2) and TargetAnalysis (version 1.3; Bruker Daltonics). Absolute amino acid quantification was based on standard curves of all analyzed amino acids between 0.25 and 5 μg. This analysis was performed using QuantAnalysis software (version 2.2; Bruker Daltonics).

### Determination of urease activity and total protein content

To measure the urease activity, shoots, and roots of maize were sampled and processed as described by Witte et al. (2001). The assay was performed using the urease activity assay kit (MAK120, Sigma Aldrich) as reported in the manufacturer's instructions. Total protein was determined using a commercial Bradford assay (Bio-Rad Laboratories, Hercules, CA) with bovine serum albumin as standard.

### Statistical analyses

Physiological and transcriptional analyses were performed on three independent biological replicates obtained from independent experiments (*n* = 3); for each sample a pool of six plants was used. Statistical significance was determined by one-way analysis of variances (ANOVA) using Student-Newman-Keuls test (*P* < 0.05, *n* = 3). Statistical analyses were performed using SigmaPlot Version 12.0 software.

Statistical analysis of microarray data was performed using linear model analysis (Smyth, 2005) of the LIMMA package after Bayesian correction with Bioconductor software, adjusted *P* ≤ 0.05, *n* = 3, *FC* ≥ |2.00| (for details see the “Microarray Analyses” Section).

## Results

### Nitrogen, urea, ammonium and protein contents in plants

In this set of experiments maize seedlings were treated for 24 h with three different nutritional conditions: nutrient solution without any source of N (*Control* treatment), nutrient solution containing urea (*Urea* treatment), nutrient solution containing urea and NBPT (*Urea*+*NBPT* treatment). No significant changes in dry weight of shoots and roots was observed among the treatments. However, already 24 h after the application of urea, changes in the N distribution were detectable. In fact plants exposed to urea, either treated with or without NBPT, accumulated higher amounts of N in shoots than in roots, while an opposite behavior was observed in *Control* plants.

Urea concentration increased both in shoots and in roots when plants were supplied with urea (*Urea* treatment), while ammonium concentration increased only in roots of these plants. Shoots of plants treated with *Urea*+*NBPT* contained significantly less N than *Urea*-treated plants (Table 1). The addition of NBPT caused a significant increase in urea concentration, this effect being especially evident in shoots. On the other hand, ammonium concentration decreased in root of *Urea*+*NBPT* treated plants with respect to *Urea*-treated plants.

**Table 1:** **Effect of NBPT on the total nitrogen (N), urea, ammonium and protein contents in shoots and roots after 24 h of treatment**.

	**Dry weight (mg plant^−1^)**	**Total *N* (mg N g^−1^ FW)**	**Urea (μmol urea g^−1^ FW)**	**Ammonium (μmol NH^+^_4_ g^−1^ FW)**	**Protein (mg prot g^−1^ FW)**
**SHOOT**					
*Control*	105.7 ± 8.1 a	10.31 ± 0.60 c	0.23 ± 0.01 c	8.43 ± 0.35 a	6.96 ± 0.38 b
*Urea*	133.7 ± 13.3 a	14.97 ± 0.14 a	0.26 ± 0.01 b	8.89 ± 0.45 a	8.19 ± 0.11 a
*Urea+NBPT*	123.4 ± 5.6 a	13.54 ± 0.33 b	0.41 ± 0.02 a	8.45 ± 0.27 a	8.89 ± 0.53 a
**ROOT**					
*Control*	110.6 ± 12 a	7.58 ± 1.14 a	0.25 ± 0.01 c	4.54 ± 0.10 b	3.52 ± 0.31 a
*Urea*	124.8 ± 4.6 a	5.28 ± 0.23 b	0.40 ± 0.03 b	6.39 ± 0.14 a	4.47 ± 0.16 a
*Urea+NBPT*	116.9 ± 4.3 a	5.18 ± 0.22 b	0.54 ± 0.01 a	4.64 ± 0.07 b	4.09 ± 0.63 a

Five-day-old maize plants were exposed for 24 h to a nutrient solution supplied with 0.5 mM urea in presence or absence of NBPT (Urea+NBPT treatment or Urea treatment, respectively) or exposed to a nutrient solution without addition of any N source (Control treatment). DW, dry weight; FW, fresh weight. Different letters indicate statistically significant differences (Student–Newman–Keuls method ANOVA, n = 3, P < 0.05).

Protein content significantly increased in shoots of plants treated with urea, irrespective of the presence of NBPT, while no significant differences among treatments were observed in root tissues (Table 1).

### Urease activity

**Figure 1:**
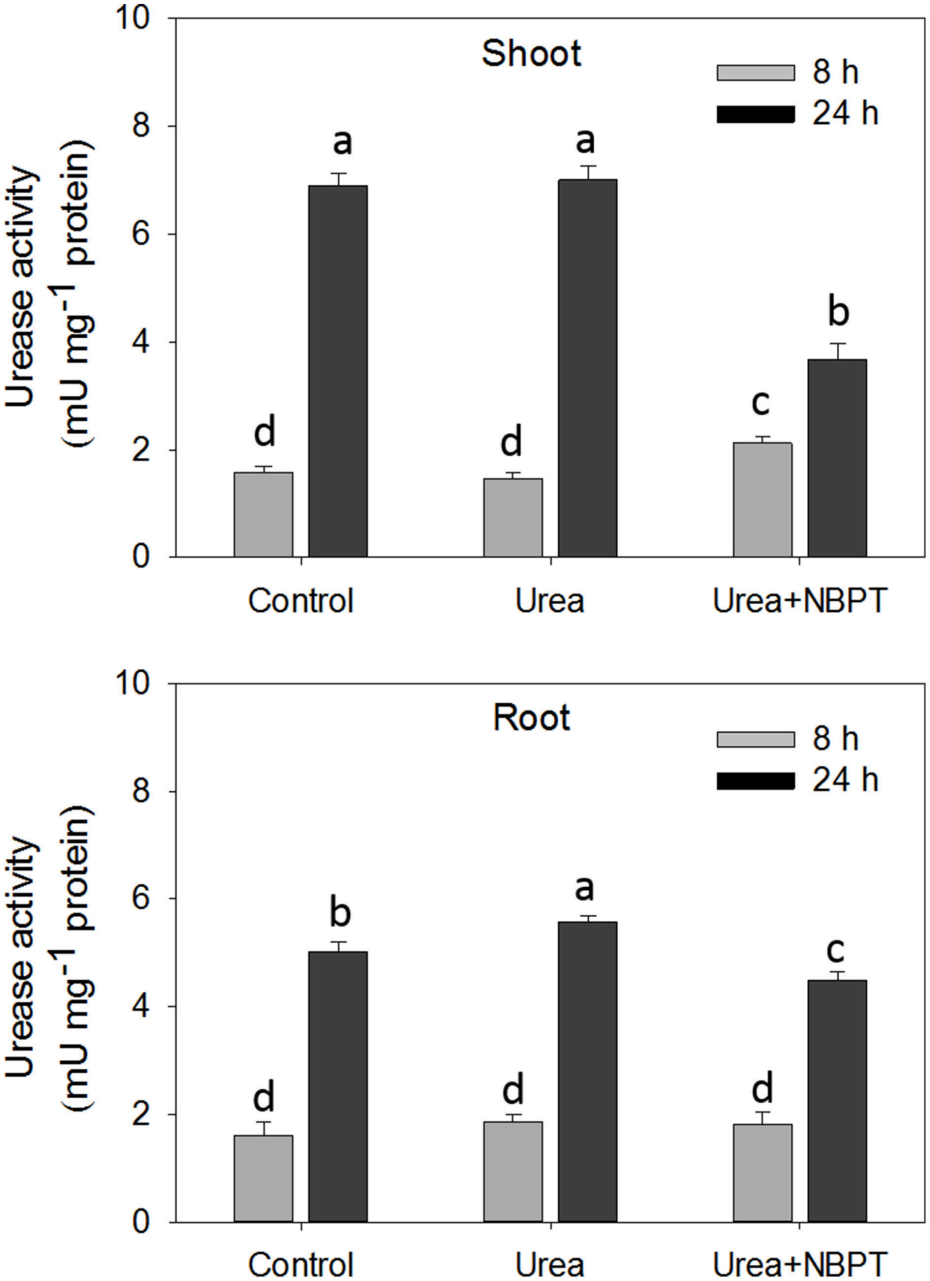
**Effect of NBPT on urease activity in shoots and roots of maize at 8 and 24 h of treatment**. Five-day-old maize plants were exposed for 24 h to a nutrient solution supplied with 0.5 mM urea in presence or absence of NBPT (*Urea*+*NBPT* treatment or *Urea* treatment, respectively) or exposed to a nutrient solution without addition of any *N* source (*Control* treatment). *DW*, dry weight; *FW*, fresh weight. Different letters indicate statistically significant differences (Student–Newman–Keuls method ANOVA, *n* = 3, *P* < 0.05).

Urease activity was measured in shoots and roots after 8 and 24 h from the beginning of the treatment. No differences in root enzyme activity was observed after 8 h of plant exposure to the different nutritional conditions (Figure 1). In shoots, only a slight increase in the enzymatic activity was observed when NBPT was added to the nutrient solution (Figure 1). Prolonging the treatment from 8 to 24 h, the ureolitic activity increased, both in shoots and roots of maize plants. This increase was significantly reduced when NBPT was added to urea containing solution, with an inhibition as high as 50% in shoots (Figure 1).

### Amino acid profiles in roots and shoots

**Figure 2:**
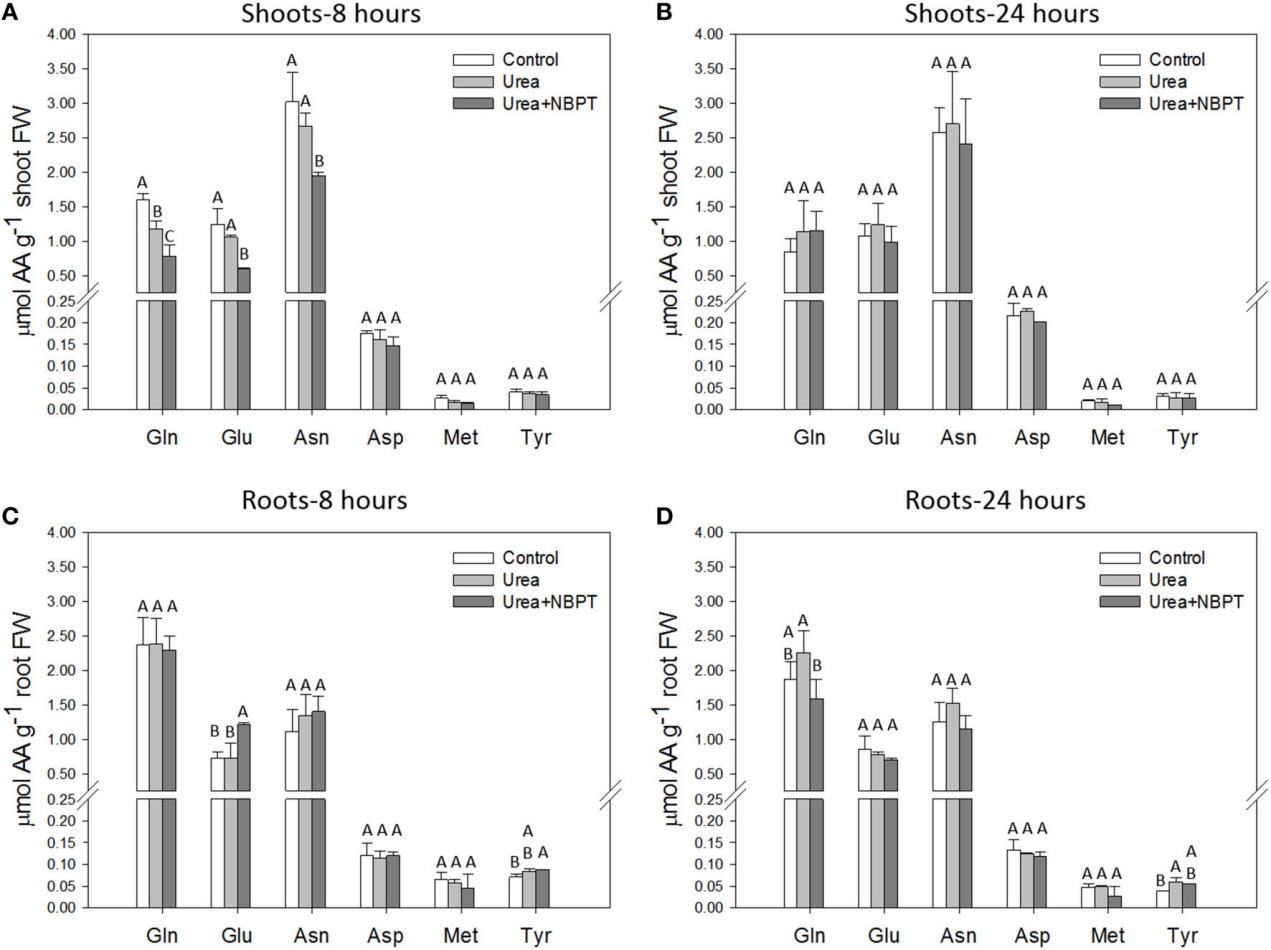
**Individual profiles of six amino acids in shoots (A,B) and roots (C,D)**. Amino acid amounts were measured on the same plants as described for Figure 1 at 8 h **(A,C)** and 24 h **(B,D)** of treatment. The values are means + *SD* of three replicates. Different letters indicate significant differences between treatments (Student–Newman–Keuls method ANOVA, *n* = 3, *P* < 0.05). AA, amino acid; FW, fresh weight.

In order to better investigate the effect of NBPT on N metabolism, we determined the concentration of six amino acids at two time points (8 and 24 h) during the nutritional treatment. The amino acids were chosen due to their key role in the primary assimilation of ureic-N (glutamine, Gln; glutamate, Glu; asparagine, Asn; aspartate, Asp) and their central function in the secondary metabolism (methionine, Met; tyrosine, Tyr). Among them, the most represented amino acids were Gln, Glu, and Asn, while the concentrations of Asp, Met, and Tyr were lower both in roots and shoots (Figure 2). Eight hours after beginning the exposure to *Urea* or *Urea*+*NBPT*, a decrease in Gln, Glu, and Asn concentrations was observed in shoots. This effect was significant in the presence of NBPT (Figure 2A). Conversely, at the same time point (8 h) the Glu levels increased in roots treated with NBPT, possibly indicating a redistribution of this amino acid within the plant (Figure 2C).

Prolonging the treatment up to 24 h, the levels of amino acids in shoots were comparable in *Control, Urea* and *Urea*+*NBPT* treated plants. Similarly, also in roots the amounts of Glu, Asn, and Asp were not affected by the treatment. On the other hand, the root concentration of Gln decreased when plants were treated with NBPT (Figure 2).

Concerning Asp, Met, and Tyr concentration no significant changes were observed among treatments. The only exception was observed for Tyr in roots of urea treated plants, since slight changes were detected at both time points when compared to *Control* roots. However, the addition of NBPT in the urea containing solution did not significantly affect the content of this amino acid (Figure 2).

### Transcriptomic changes induced by NBPT in roots of urea-fed plants

To evaluate if physiological changes could be linked to modulations of gene expression, transcriptomic analyses were performed in maize roots after exposure for 8 h to *Urea*+*NBPT* or *Urea* treatment. Differentially expressed genes were identified by Linear Models for MicroArray (LIMMA, Smyth, 2005), as the subset of genes displaying at least a 2-fold change in transcript abundance (*FC* ≥ |2.00|, *P* ≤ 0.05). The analyses revealed that when NBPT was added to the nutrient solution, 1002 transcripts were significantly upregulated, while 1624 were downregulated, Supplementary Table 4).

**Figure 3:**
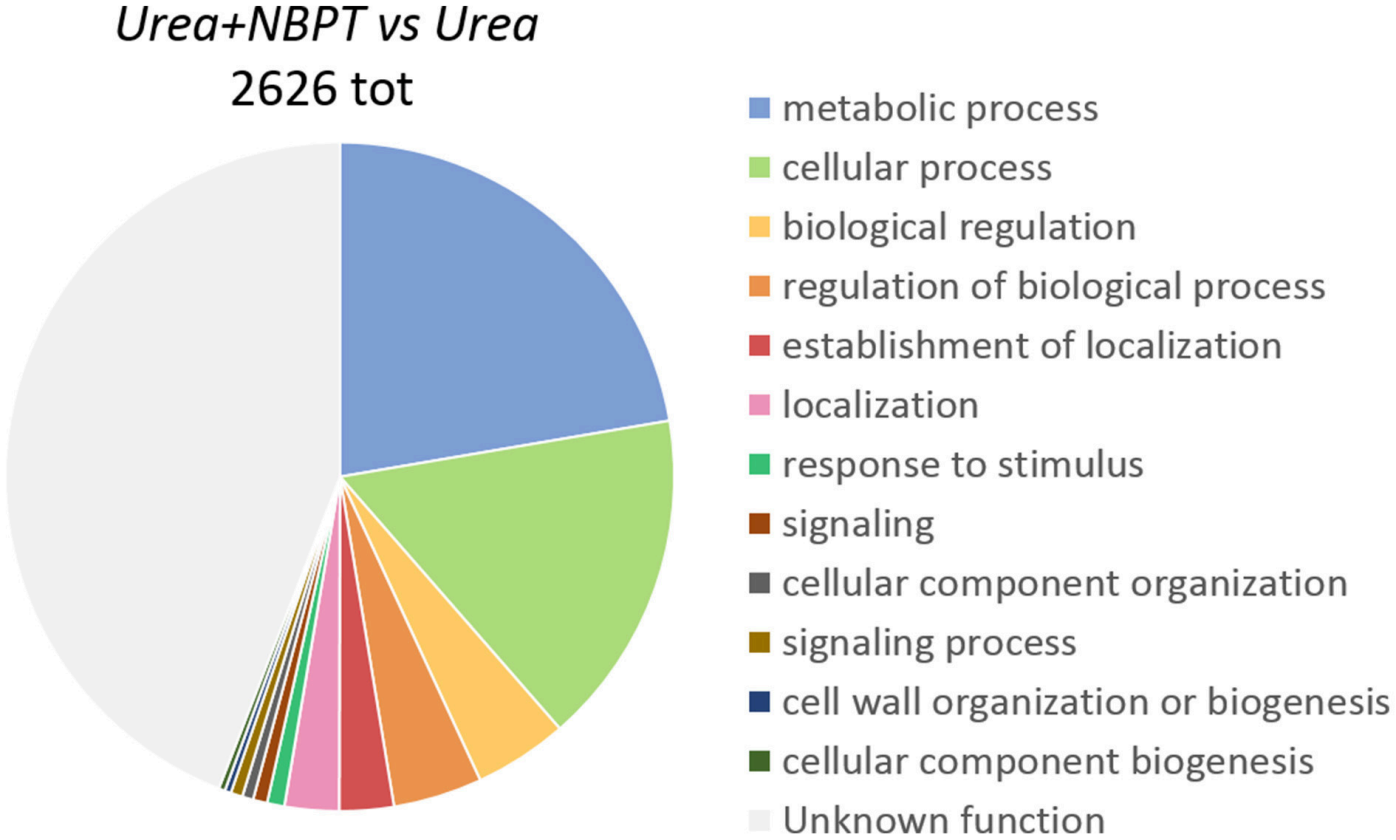
**Functional category distribution of differentially expressed transcripts in *Urea*+*NBPT* vs. *Urea*, according to the terms of biological process of Gene Ontology (GO)**.

**Figure 4:**
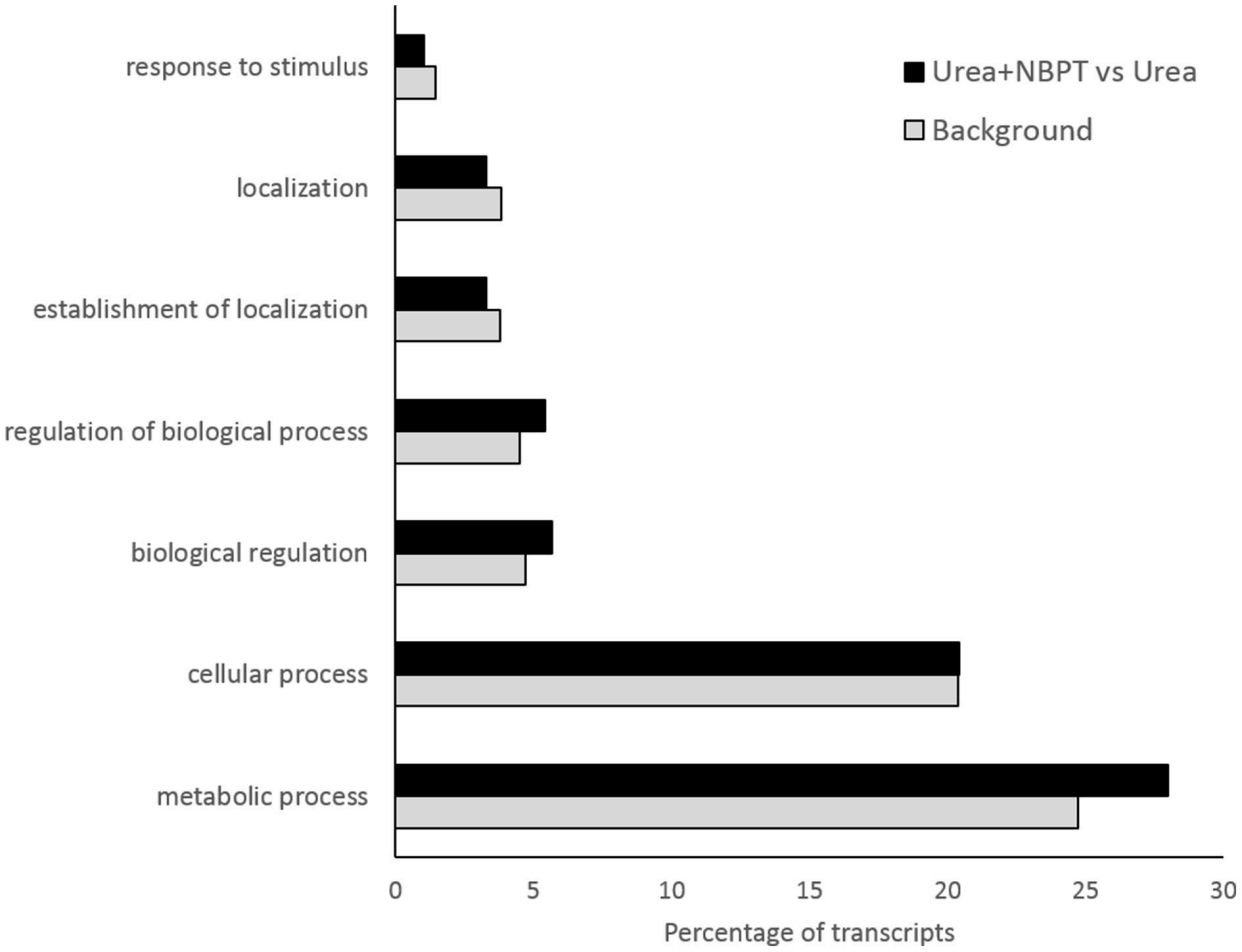
**GO-term enrichment analysis of the significantly regulated genes in *Urea*+*NBPT* vs. *Urea***. Significantly most enriched GO-terms for biological process GOs are plotted according to increasing enrichment of the percentage of genes in the data set of *Urea*+*NBPT* vs. *Urea* (black bars) compared to that of the maize reference set (*Background,* gray bars). Biological Process GOs—GO:0050896 response to stimulus, GO:0051179 localization, GO:0051234 establishment of localization, GO:0050789 regulation of biological process, GO:0065007 biological regulation, GO:0009987 cellular process, GO:0008152 metabolic process. (SEA analysis using AgriGO toolkit: hypergeometric tests with Yekutieli as multi-test adjusted method, *P* < 0.05).

According to the terms of biological processes of Gene Ontology (GO) the most representative functional categories in the *Urea*+*NBPT* vs. *Urea* comparison were “metabolic process,” “cellular process,” “biological regulation,” “regulation of biological process,” “establishment of localization,” “localization,” and “response to stimulus” (Figure 3). The GO categories “Metabolic process,” “biological regulation,” and “regulation of biological process” were found to be enriched in the *Urea*+*NBPT* vs. *Urea* comparison with respect to the whole maize transcriptome (used as background reference for Singular Enrichment Analyses, SEA Figure 4). On the other hand “signaling,” “cellular component organization,” “signaling process,” “cell wall organization and biogenesis,” and “cellular component biogenesis” categories were less abundantly represented in the data set *Urea*+*NBPT* vs. *Urea* (< 1%, Figure 3).

**Figure 5:**
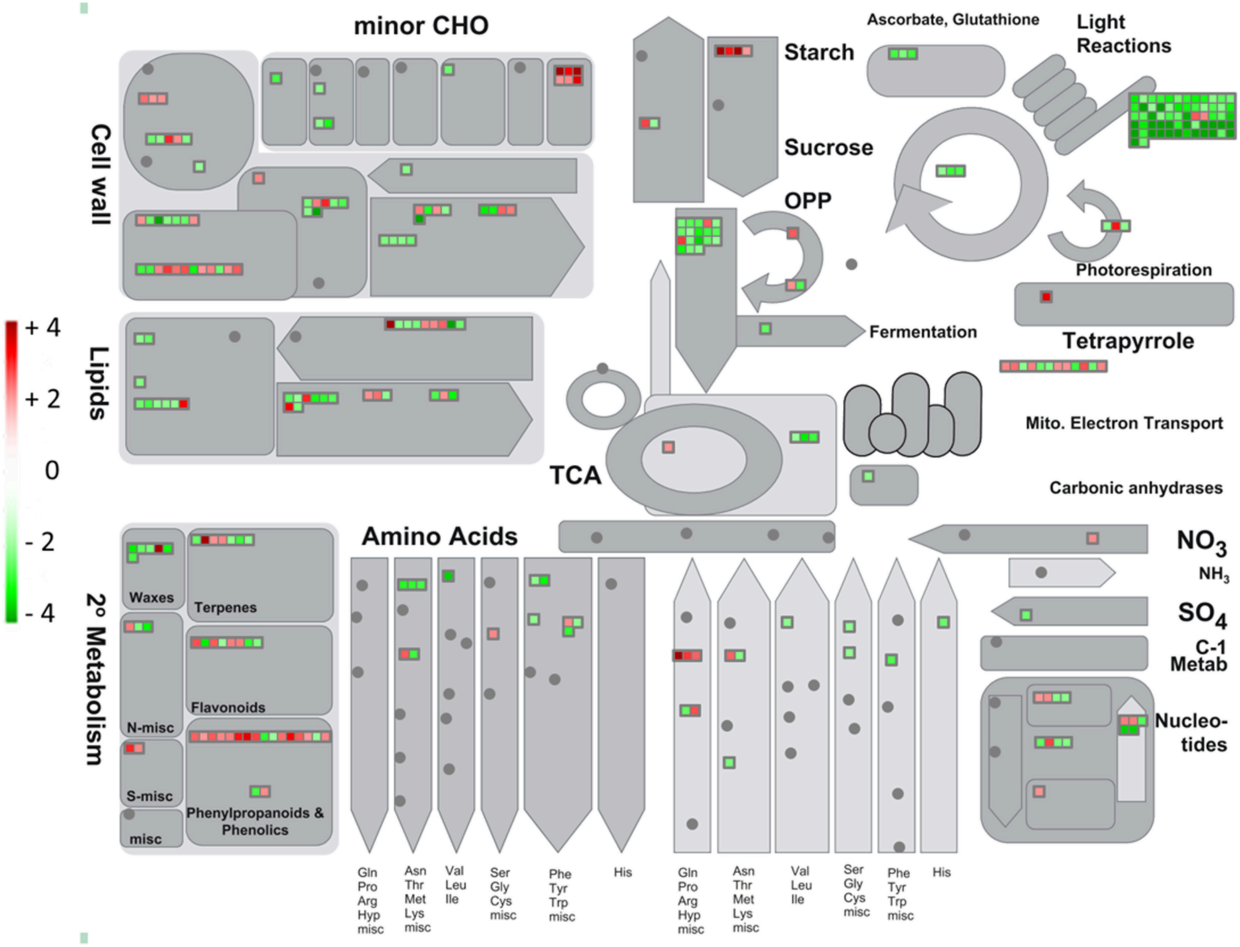
**Transcriptional modulation of genes involved in the cell metabolism in *Urea*+*NBPT* vs. *Urea* roots**. Color scale refers to the fold change values of differentially expressed transcripts: *red* color refers to those transcripts positively regulated by *Urea*+*NBPT* treatment, while in *green* are transcripts negatively regulated by *Urea*+*NBPT* treatment.

The analyses of the genes involved in the metabolic, regulatory and transport pathways might allow improving the comprehension of physiological changes occurring in NBPT-treated plants. Therefore, to identify which pathways were affected by the NBPT treatment, up- and down-regulated transcripts were classified into different groups according to the bin divisions of the MapMan software (Version 3.6.0RC1; Thimm et al., 2004; Figure 5, Supplementary Figure 3).

**Figure 6:**
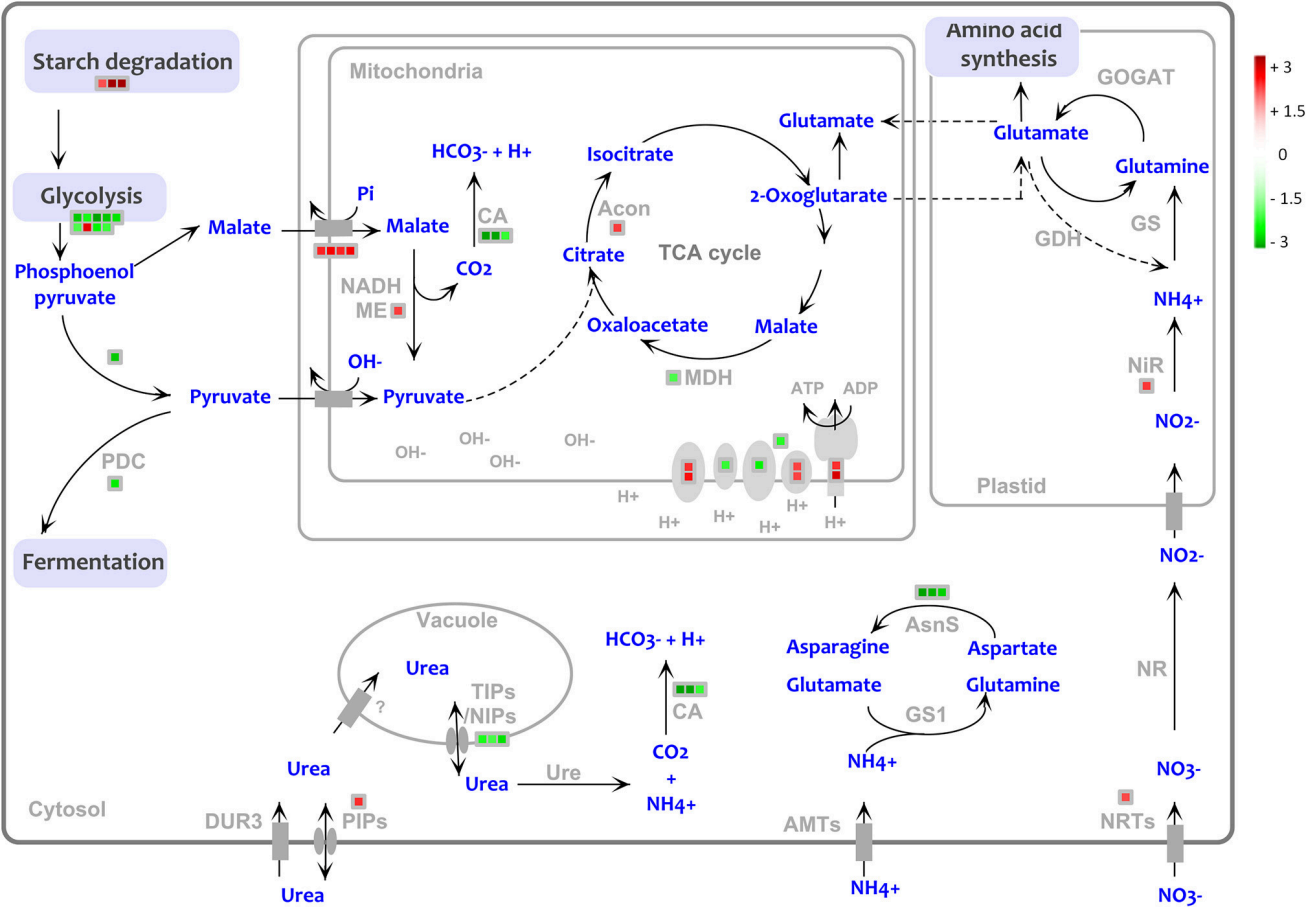
**Transcriptional changes of genes involved in the urea assimilation and primary metabolism in *Urea*+*NBPT* vs. *Urea* roots**. PDC, pyruvate decarboxylase; NADH-ME, NADH malic enzyme, MDH, malate dehydrogenase, CA, carbonic anhydrase; Acon, aconitase; GOGAT, glutamate oxoglutarate aminotransferase, GDH, glutamate dehydrogenase; NiR, nitrite transferase; NR, nitrate reductase; NRTs, nitrate transporters; AMTs, ammonium transporters; GS1, glutamine synthetase 1; AsnS, asparagine synthetase; Ure, urease; PIPs, plasma membrane intrinsic proteins; TIPs/NIPs, tonoplast intrinsic protein/ NOD26-like intrinsic protein; DUR3, high affinity urea transporter. Color scale refers to the fold change values of differentially expressed transcripts: *red* color refers to those transcripts positively regulated by *Urea*+*NBPT* treatment, while in *green* are transcripts negatively regulated by *Urea*+*NBPT* treatment.

Some genes involved in the pathway of urea acquisition were found to be modulated by the presence of NBPT (Figure 6). Compared to *Urea* roots, the treatment with *Urea*+*NBPT* upregulated a gene coding for urease (# 202 GRZM2G461569_T03, Table 2). This is an alternative transcript of the *urease* gene and covers only 20% of the complete coding sequence (primary *urease* transcript GRZM2G461569_T01). Thus, it appears that this truncated mRNA (# 202) encodes a non-functional urease (for functional annotation see: www.phytozome.jgi.doe.gov).

**Table 2:** **List of modulated transcripts involving in urea acquisition and reported in the *Results* and *Discussion* Sections by the comparison of transcriptional profiles of *Urea*+*NBPT* roots with profile of *Urea* roots (*Urea*+*NBPT* vs. *Urea* comparison)**.

**#**	**Transcript ID**	***FC***	***P*-value adj**.	**Description**	**Short name**
93	GRMZM2G348512_T04	–3.82	0.005	*Carbonic anhydrase*	*CA*
94	GRMZM2G348512_T03	–3.09	0.013	*Carbonic anhydrase*	*CA*
95	GRMZM2G009633_T04	–2.15	0.004	*Carbonic anhydrase*	*CA*
183	GRMZM2G079381_T04	2.14	0.017	*Nitrite reductase 1*	*NiR1*
185	GRMZM2G313272_T01	–3.02	0.015	*Glutamine-dependent asparagine synthase 1*	*ASN1*
186	GRMZM2G078472_T02	–2.90	0.018	*Glutamine-dependent asparagine synthase 1*	*ASN1*
187	GRMZM2G078472_T04	–2.72	0.005	*Glutamine-dependent asparagine synthase 1*	*ASN1*
202	GRMZM2G461569_T03	2.66	0.017	*Urease*	*URE*
543	GRMZM2G001205_T01	–2.50	0.004	*Zinc finger (C2H2 type) protein*	*ZFP16-1*
1283	GRMZM2G024808_T01	2.05	0.017	*MFS nitrate and chloride transporter*	*MFS*
1295	GRMZM2G458494_T01	2.73	0.008	*MFS nitrate and chloride transporter*	*MFS*
1338	GRMZM2G375113_T01	2.04	0.039	*MFS nitrate and chloride transporter*	*MFS*
1355	GRMZM2G014914_T03	2.20	0.009	*Plasma membrane intrinsic protein 2*	*PIP2;2*
1356	GRMZM2G146627_T01	–2.30	0.008	*Tonoplast intrinsic protein 4;1*	*TIP4;1*
1357	GRMZM5G892338_T02	–2.05	0.020	*NOD26-like intrinsic protein 4;2*	*NIP4;2*
1358	GRMZM5G892338_T01	–2.61	0.002	*NOD26-like intrinsic protein 4;2*	*NIP4;2*

In the table are shown (starting from left column): number of transcript (see Supplementary Table 1), transcript ID, fold change (FC) of Urea+NBPT vs. Urea, P-value adjusted, transcript description and short name were reported for each transcript.

Our results also show that several genes closely related to urea assimilation are downregulated: three transcripts coding for carbonic anhydrases (# 93–95, Table 2), three alternative transcript coding for a glutamine-dependent asparagine synthetase (# 185–187, Table 2) and a transcription factor ZFP16-1 that has been reported to be early responsive to urea nutrition (# 543, Table 2). A similar trend was also observed for some genes coding for aquaporins (nodulin 26-like intrinsic protein, NIP, and tonoplast intrinsic proteins, TIPs, # 1356-1358, Table 2) that might be involved in the low affinity transport of urea. On the other hand, one aquaporin that is putatively localized in the plasma membrane (# 1355 Table 2), as well as transcripts coding for nitrate transport and assimilation (# 183, 1283, 1295, 1338, Table 2), were upregulated by *Urea*+*NBPT* (Figure 6).

As regards primary metabolism, a considerable downregulation was observed for genes involved in glycolysis and fermentation (PFK, enolase, G-6P isomerase, pyruvate decarboxylase and a putative phosphoenolpyruvate carboxykinase, GRMZM2G580389, # 77–83, 85, and 1704, Supplementary Table 4) as well as in the TCA cycle (ATP citrate lyase, malate dehydrogenase, carbonic anhydrase, # 90–95, Supplementary Table 4, Figures 5, 6). A similar expression pattern was also observed for genes related to the photosynthetic pathway, including light reactions (# 1–54, Supplementary Table 4), photorespiration (# 55–57, Supplementary Table 4), and Calvin cycle (RubisCo, TPI, FBPase, # 58–60, Supplementary Table 4, Figure 5). This modulation was further supported by a downregulation of some plastidic triose-phosphate translocators (TPTs, # 1318–1319, Supplementary Table 4), indicating a crosslink between Calvin cycle and glycolysis.

Despite glycolysis was negatively affected by NBPT, the upstream reactions for the starch degradation (beta-amylase, heteroglycan glucosidase, # 63–65, Supplementary Table 4) were induced in *Urea*+*NBPT* roots. In a similar way, also two genes encoding for key enzymes of TCA cycle (aconitase and NADH-malic enzyme, # 88–89, Supplementary Table 4) and one for the translocation of malate into mitochondria (dicarboxylated transporters, # 1324–1325, Supplementary Table 4) were upregulated (Figure 6).

A consistent modulation was also detected for genes related to the electron transport chain: the expression of six transcripts were upregulated by the NBPT treatment (NADH dehydrogenase, cytochrome c oxidase, and ATP synthase, # 96–97, 101–102, 105–106, Supplementary Table 4), while three genes coding for cytochrome c reductase, a cytochrome c, and a flavoprotein were found to be downregulated (# 98–100, Supplementary Table 4, Figure 6).

Concerning the secondary metabolism, some genes involved in arginine catabolism and polyamine pathway were also modulated. In particular a gene coding for an arginine decarboxylase (ADC1, # 201, Supplementary Table 4) was downregulated by *Urea*+*NBPT*, as well as several other genes encoding enzymes for polyamine degradation: polyamine oxidase (PAO1, PAO3) and copper amine oxidase (PAO and CuAO, # 372–373, 437, 1499, and 205, Supplementary Table 4). Along with putrescine, S-adenosyl methionine (SAM) participates as methyl donor to the synthesis of spermidine and spermine, a particular class of polyamines. The treatment with *Urea*+*NBPT* led to the upregulation of a gene for the synthesis of SAM (# 188, Supplementary Table 4), but limited the expression of some SAM methyltransferases (# 189, 343–344, 1534, 1568, 2048, Supplementary Table 4), putatively involved in other SAM-consuming pathways. Beside these changes linked to arginine catabolism, results showed that three transcripts encoding proline oxidase (# 198–200, Supplementary Table 4) were upregulated by *Urea*+*NBPT*.

**Figure 7:**
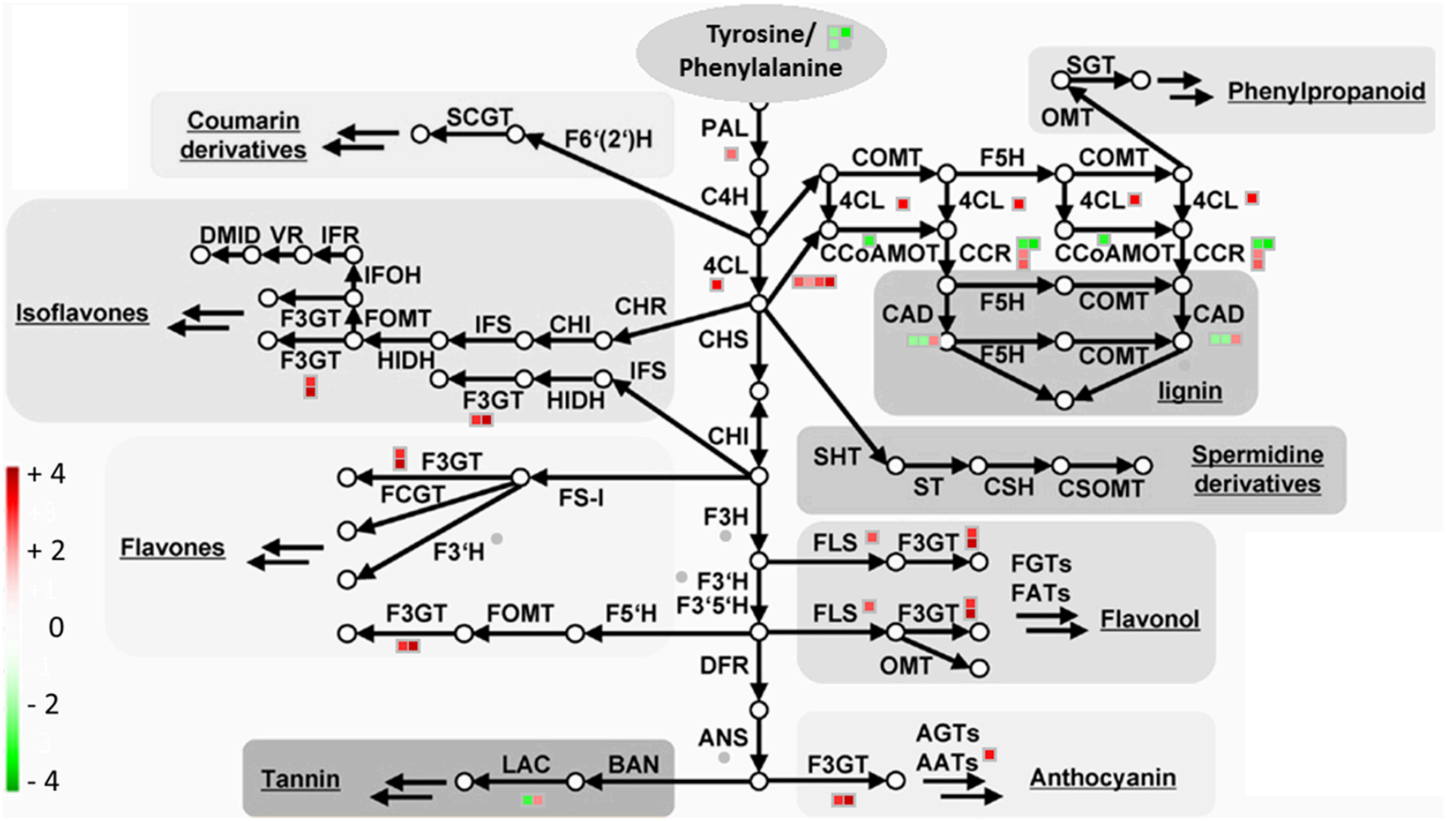
**Transcriptional modulation of genes involved in the phenylalanine/tyrosine derived secondary metabolite biosynthesis in *Urea*+*NBPT* vs. *Urea* roots**. In maize plants, phenylalanine, and tyrosine are precursor for the synthesis of several secondary metabolites. Color scale refers to the fold change values of differentially expressed transcripts: *red* color refers to those transcripts positively regulated by *Urea*+*NBPT* treatment, while in *green* are transcripts negatively regulated by *Urea*+*NBPT* treatment. PAL, phenylalanine ammonia-lyase; C4H, cinnamate-4-hydroxylase; 4CL, 4-coumarate CoA ligase; CAD, cinnamoyl-alcohol dehydrogenase; F5H, ferulate 5-hydroxylase; C3H, coumarate 3-hydroxylase; ALDH, aldehyde dehydrogenase; CCR, cinnamoyl-CoA reductase; HCT, hydroxycinnamoyl-Coenzyme A shikimate/quinate hydroxycinnamoyltransferase; CCoAOMT, caffeoyl/CoA-3-O-metheltransferase; CHS, chalcone synthese; CHI, chalcone isomerase; F3H, flavanone 3-hydroxylase; F3′H, flavonoid-3′-hydroxylase; F3GT, flavonoid-3-O-glycosyltransferase; FS, flavone synthase; FOMT, flavonoid O-methyltransferase; FCGT, flavone-C-glycosyltransferase; FLS, flavonol synthese; F3GT, flavonoid-3-O-glycosyltransferase; DFR, dihydroflavonol reductase; ANS, Anthocyanidin synthese; AGT, Flavonoid-O-glycosyltransferase; AAT, anthocyanin acyltransferase; BAN, oxidoreductase|dihydroflavonol reductase like; LAC, laccase. The image is adapted from Tohge et al. (2013).

Another set of transcripts involved in the secondary metabolism was related to the synthesis of phenylpropanoids (# 193–195, 225, 227, 232–238, 250–258, 289, 412, Table 3, Figure 7). In particular, the NBPT treatment downregulated the expression of three transcripts related to the synthesis of shikimate and arogenate (# 193–195, Table 3), while the downstream reactions for the synthesis of phenylpropanoids were upregulated (# 227, 232–235, 237–238, 250–251, 255–256, 258, 289, 412, Table 3), with the exception of some transcripts involved in the lignin synthesis, that were downregulated (# 225, 236, 252–254, Table 3).

**Table 3:** **List of modulated transcripts involving in the phenylpropanoid synthesis and reported in the *Results* and *Discussion* sections by the comparison of transcriptional profiles of *Urea*+*NBPT* roots with profile of *Urea* roots (*Urea*+*NBPT* vs. *Urea* comparison)**.

**#**	**Transcript ID**	***FC***	***P*-value adj**.	**Description**	**Short name**
193	GRMZM2G454719_T01	–2.13	0.003	*Phospho-2-dehydro-3-deoxyheptonate aldolase (DAHP synthetase)*	*DAHP-S*
194	GRMZM2G036861_T05	–3.21	0.002	*Chorismate synthase*	*CS*
195	GRMZM2G429057_T01	–2.08	0.008	*Pyridoxal phosphate (PLP)-dependent aminotransferase*	*PAT*
225	GRMZM2G104710_T01	–2.91	0.030	*Caffeate O-methyltransferase, putative*	*CCoAMT*
227	GRMZM2G107211_T02	2.04	0.048	*Hydroxycinnamoyl-CoA shikimate/quinate hydroxycinnamoyl transferase*	*HCT*
232	GRMZM2G035584_T07	2.62	0.007	*Hydroxycinnamoyl-CoA shikimate/quinate hydroxycinnamoyl transferase*	*HCT*
233	GRMZM2G156816_T04	2.73	0.003	*Hydroxycinnamoyl-CoA shikimate/quinate hydroxycinnamoyl transferase*	*HCT*
234	GRMZM2G175082_T01	3.52	0.006	*Hydroxycinnamoyl-CoA shikimate/quinate hydroxycinnamoyl transferase*	*HCT*
235	GRMZM2G074604_T03	2.40	0.004	*Phenylalanine ammonia-lyase*	*PAL2*
236	GRMZM2G167613_T01	–2.05	0.006	*Cinnamyl alcohol dehydrogenase 9*	*CAD9*
237	GRMZM2G700188_T05	2.21	0.005	*Cinnamyl alcohol dehydrogenase 7*	*CAD7*
238	GRMZM2G054013_T04	3.27	0.003	*4-coumarate-CoA ligase*	*4CL2*
250	GRMZM5G882427_T01	3.12	0.024	*Anthocyanin 5-aromatic acyltransferase 1*	*AAT1*
251	GRMZM2G015709_T01	3.72	0.006	*Flavonol 3-O-glucosyltransferase*	*F3GT*
252	GRMZM2G179685_T04	–3.34	0.008	*Cinnamoyl-CoA reductase-related*	*CCR*
253	GRMZM2G179685_T05	–2.85	0.023	*Cinnamoyl-CoA reductase-related*	*CCR*
254	GRMZM2G146623_T02	–2.03	0.037	*Cinnamyl-alcohol dehydrogenase*	*CAD*
255	GRMZM2G179685_T01	2.25	0.035	*Cinnamoyl-CoA reductase-related*	*CCR*
256	GRMZM2G131205_T06	2.57	0.034	*Cinnamoyl-CoA reductase*	*CCR1*
257	GRMZM5G870184_T01	–2.77	0.019	*Laccase-15 precursor*	*LAC7*
258	GRMZM2G166857_T01	2.15	0.020	*Laccase-15 precursor*	*LAC7*
289	GRMZM2G058024_T02	2.66	0.018	*Flavonol synthase*	*FLS*
412	GRMZM2G022266_T01	2.92	0.003	*Flavonol 3-O-glucosyltransferase*	*F3GT*

In the table are shown (starting from left column): number of transcript (see Supplementary Table 4), transcript ID, fold change (FC) of Urea+NBPT vs. Urea, P-value adjusted, transcript description and short name were reported for each transcript.

The presence of NBPT in the root external solution also modulated the expression of genes related to biotic (# 305–332, Supplementary Table 4) and abiotic stresses (# 333–351, Supplementary Table 4), this latter group of transcripts being mainly downregulated by NBPT. As a general response to a stress condition we observed the induction of some genes involved in callose synthesis (glucan synthases, # 71–75, Supplementary material Supplementary Table 4) and coding for cell wall proteins (AGPs and LRRs, # 114–115 and 117–118, Supplementary Table 4). On the other hand some genes related to cell wall degradation (xylosidase and glucanases, # 119–124, Supplementary Table 4) and modification (pectinesterases, # 145–149, Supplementary Table 4) were downregulated by NBPT (Figure 5).

This transcriptional behavior might be closely related to the hormonal response of root cells. Indeed some genes linked to auxin (# 263–274, Supplementary Table 4) and ethylene metabolism (# 283–295, Supplementary Table 4) were mostly negatively modulated in *Urea*+*NBPT* treated root cells. A different behavior was observed for genes related to jasmonic acid biosynthesis (# 298–301, Supplementary Table 4), which were upregulated by the treatment with the urease inhibitor.

Finally, a miscellaneous group of transcripts, mainly coding for transporters and transcription factors involved in the acquisition of other nutrients, was found to be modulated by NBPT treatment. Most of them were related to the acquisition and homeostasis of Fe. Indeed two transcripts coding for putative Fe(III)-PS transporters (ZmYSL7, # 1333, 1335; Table 4), one coding for the vacuolar storage of Fe (VIT1, # 219; Table 4), one coding for the plastid protein uptake machinery (TIC21, # 868; Table 4) and some transcripts coding for bHLH transcription factors (ZmPYE-like, # 673–674, 2026 and ZmbHLH29, # 684; Table 4) were modulated by the *Urea*+*NBPT* treatment.

**Table 4:** **List of modulated transcripts involving in the acquisition of other nutrients and reported in the *Results* and *Discussion* sections by the comparison of transcriptional profiles of *Urea*+*NBPT* roots with profile of *Urea* roots (*Urea*+*NBPT* vs. *Urea* comparison)**.

**#**	**Transcript ID**	***FC***	***P*-value adj**.	**Description**	**Short name**
219	GRMZM2G074672_T02	6.47	0.001	*Vacuolar iron transporter 1*	*VIT1*
673	GRMZM2G017145_T03	–2.54	0.002	*Basic helix-loop-helix (bHLH)*	*PYE-like*
674	GRMZM2G017145_T02	–2.48	0.002	*Basic helix-loop-helix (bHLH)*	*PYE-like*
684	GRMZM2G313756_T01	2.26	0.049	*Basic helix-loop-helix (bHLH) FER-like regulator of iron uptake*	*bHLH29-like*
868	GRMZM2G145500_T04	–2.70	0.004	*Copper/iron translocation at inner membrane of chloroplast*	*TIC21*
1333	GRMZM2G120922_T01	–3.15	0.007	*Yellow stripe like 7*	*YSL7*
1335	GRMZM2G062844_T01	–2.34	0.026	*Yellow stripe like 7*	*YSL7*
1361	GRMZM5G870170_T01	–3.14	0.002	*MATE type citrate carrier*	*MATE1*
2026	GRMZM2G017145_T05	–2.18	0.013	*Basic helix-loop-helix (bHLH)*	*PYE-like*
2144	GRMZM2G161310_T02	–2.07	0.010	*Zinc induced facilitator 1*	*ZIF1*

In the table are shown (starting from left column): number of transcript (see Supplementary Table 4), transcript ID, fold change (FC) of Urea+NBPT vs. Urea, P-value adjusted, transcript description and short name were reported for each transcript.

Exposure of maize roots to *Urea*+*NBPT* influenced also the expression of genes putatively involved in micronutrient complexation: a gene encoding a putative nicotianamine transporter (*ZmZIF1*, # 2144; Table 4) and a gene mediating the efflux of citrate into the rhizosphere (*ZmMATE1*, # 1361; Table 4) were negatively affected by *Urea*+*NBPT*.

## Discussion

N-(n-butyl) thiophosphoric triamide (NBPT) is the most widely used urease inhibitor (Sanz-Cobena et al., 2012; Abalos et al., 2014). However, only few information is available on the effect of NBPT on N acquisition mechanisms in plants.

An inhibitory effect of NBPT on urea uptake and assimilation was shown in a previous work (Zanin et al., 2015a). In the present work, a transcriptomic analysis was performed in order to highlight the modifications induced by a short-term (8 h) treatment with NBPT in the primary and secondary metabolism of urea-fed maize seedlings.

### Urea and ammonium assimilation pathways

It is well-known that the acquisition of external urea is mediated by several proteins localized on the root plasma membrane. A specific transporter, DUR3, and some members belonging to the aquaporin family are considered to be the major components of the high- and low-affinity-transport system in plants, respectively (for review see Kojima et al., 2006).

Confirming previous evidence (Zanin et al., 2015a), transcriptomic analysis revealed that the expression of *DUR3* gene was not affected by the NBPT treatment, while a different behavior was observed for several aquaporins. Among the modulated transcripts, one sequence (*ZmTIP4;1,* # 1356 Table 2) exhibited a high homology to *AtTIP4;1* that encodes a tonoplastic aquaporin of arabidopsis permeable to urea and inducible by N deficiency in roots (Liu et al., 2003). When the urease inhibitor NBPT was added to the external solution, root cells accumulated urea (Table 1); this in turn would modulate positively or negatively the expression of different aquaporins (# 1355–1358, Table 2) in order to equilibrate the concentration of urea within different cellular compartments (Figure 6).

In the cytosol, urea should be rapidly hydrolyzed by urease, releasing ammonium and carbonic dioxide but, as reported in wheat and pea plants (Artola et al., 2011; Cruchaga et al., 2011), the treatment with the urease inhibitor NBPT interferes with the assimilation of urea and decreases plant urease activity. Also in maize, the NBPT treatment compromised the urease activity (Figure 1).

After 8 h of treatment, the urea concentration already increased in roots (Zanin et al., 2015a), although the enzyme activity was not yet affect by NBPT (Figure 1). At the same time, microarray analyses revealed that the *Urea*+*NBPT* treatment positively modulates the expression of a truncated transcript (5′-smallRNA) putatively coding for a partial and inactive fragment of urease (# 202 Table 2), while the expression of the complete transcript coding for a functional urease was not influenced by NBPT (Zanin et al., 2015a). In response to changes in urea concentration, plant cells might regulate the urease activity through a transcriptional attenuation mechanism, as reported in bacteria. In fact, in *Helicobacter pylori*, a urease-regulatory mechanisms mediated by 5′-smallRNA downregulates the urease expression through a premature termination of the transcript (Wen et al., 2013).

According to the proposed pathway for ureic-N assimilation (Mérigout et al., 2008; Pinton et al., 2016), ammonium derived from urea catabolism is rapidly assimilated by a cytosolic pathway involving a glutamate dehydrogenase, a glutamine synthase and an asparagine synthetase. In the presence of *Urea*+*NBPT*, the root capability to hydrolyze urea (Figure 1) decreased reducing the amount of ammonium available for the primary assimilation (Table 1); this result is confirmed by the low amounts of Gln measured in the roots (Figure 2D). Consistent with these observations, the microarray experiment identified the downregulation of genes encoding asparagine synthetase and carbonic anhydrase (# 185–187 and 93–95, Table 2, Figure 6), two enzymes consuming the ureolitic products (ammonium and carbon dioxide). Furthermore, after 8 h of treatment, the amounts of Glu, Gln, and Asn in shoots were reduced by NBPT (Figure 2A); in roots, Glu content increased while Gln or Asn did not exhibit any significant change (Figure 2C). The root accumulation of Glu supports the idea that NBPT might limit ureic-N assimilation. It is plausible that the NBPT treatment would determine a rapid redistribution of amino acids from shoots to roots.

Other genes involved in N acquisition were modulated when plants were treated with *Urea*+*NBPT*. Since the nutrient solution did not contain nitrate and no measurable urea degradation occurred in the external medium, the upregulation of genes coding for putative nitrate transporters and for nitrite reductase (# 1283, 1295, 1338, 183, Table 2, Figure 6) might be activated to compensate for the reduced N assimilation. All these changes involved in the urea assimilation pathway might be triggered by the action of the transcription factor ZFP16-1 (# 543, Table 2), a homolog to ZAT12 in arabidopsis, whose gene expression is known to be responsive to urea nutrition (Mérigout et al., 2008; Zanin et al., 2015b) and to abiotic stresses (Davletova et al., 2005). As an early responsive element (Zanin et al., 2015a), this transcription factor might play a key role in activating the pathway for urea assimilation and in turn the inducible acquisition of urea in plants. The downregulation of *ZFP16-1* by NBPT might limit the overall mechanism of ureic-N assimilation and redistribution in plants.

### Primary metabolism

In roots of *Urea*+*NBPT* treated plants, many genes involved in primary metabolic pathways, like glycolysis and TCA cycle (# 77–83, 85, 90–95, and 1704, Supplementary Table 4, Figures 5, 6), were downregulated. However, a few transcripts, such as malate/dicarboxylate transporters (for the mitochondrial translocation of malate), a NADPH-malic enzyme and aconitase, were induced by NBPT (# 88–89, 1324–1325, Supplementary Table 4). Aconitase converts citrate to isocitrate, a precursor of α-ketoglutarate, which is a key metabolite for N assimilation. These modulations in transcript levels might indicate the activation of an alternative pathway to sustain the TCA cycle through translocation of cytosolic malate into mitochondria *via* an antiport with Pi, rather than via a pyruvate/OH^−^ antiport (Taiz and Zeiger, 2002, Figure 6). In this way, the dissipation of the transmembrane pH gradient through the mitochondria inner membrane is avoided. Moreover, in the matrix, malate becomes the substrate of malic enzyme which generates pyruvate and, in turn, participates (as acetyl-CoA) in the TCA cycle. The decarboxylation of malate leads to the consumption of H^+^ and to the release of a CO_2_ molecule (Figure 6). This alkalization could be potentially compensated by the transformation of CO_2_ into bicarbonate, a reaction catalyzed by carbonic anhydrases. However, the transcriptomic analyses revealed that these latter enzymes were downregulated by NBPT (# 93–95, Supplementary Table 4, Figure 6), as a possible response of root cells to preserve the alkalization of mitochondrial matrix.

Thus, in the presence of *Urea*+*NBPT*, root cells seem to activate an alternative pathway to limit mitochondrial alkalization of the intermembrane space and, at the same time, to increase the pH of the matrix, as a mechanism to enhance the transmembrane potential suitable for ATP synthesis. In fact there were some positive modulations in the expression of genes coding for mitochondrial proteins involved in ATP synthesis and electron transport (complex I and IV). This response might be a strategy to compensate the wide downregulation occurring in the plastidial electron transport chain (# 1–54, Supplementary Table 4, Figure 6).

### Secondary metabolic pathway: changes triggered by the urease inhibitor NBPT

#### Arginine and polyamine synthesis

Urea, either derived from the rhizosphere or from internal N cycling (*via* arginine catabolism), plays an important role as N metabolite in plants (Witte, 2011). In addition to its role in urea synthesis, arginine is a versatile amino acid and serves as a precursor for the synthesis of polyamines, glutamate, proline and participates also in the biosynthesis of several alkaloids (nicotine, tropane-, and pyrrolidine-alkaloids; Sato and Yamada, 2008).

It is known that the polyamine (PA) homeostasis in plants correlates with several important physiological functions, including the control of the C:N ratio (Mattoo et al., 2006), stress response (Alcázar et al., 2011) and protein regulation (Baron and Stasolla, 2008; Tisi et al., 2011a,b). To understand if the plant response to the urease inhibitor NBPT might have consequences on N cycling, we extracted from the gene list the transcripts encoding proteins that are related to arginine and PAs metabolism. So far two different genes coding for isoforms of arginine decarboxylase (*ADC1* and *ADC2*), the first enzyme for the polyamine synthesis, have been identified in the plant genome. The arginine decarboxylation is a limiting step for PA synthesis; in fact the overexpression of ADC2 enzyme isoform has been shown to promote putrescine accumulation (Alcázar et al., 2005). Our transcriptomic data revealed a downregulation for *ADC1* (*ADC1* # 201, Supplementary Table 4) but not for *ADC2* when plants were treated with NBPT. These two enzymatic isoforms show different expression patterns depending on the nature of the stress and appear to play specific roles in response to different stresses (Alcázar et al., 2010). While the expression of *ADC1* is highly responsive to cold conditions, *ADC2* is induced by a wide range of stresses, such as drought, wounding/jasmonate treatment, salinity, and potassium deficiency (Urano et al., 2003; Cuevas et al., 2008; Alcázar et al., 2010). Moreover, the arabidopsis homolog of the urea-responsive transcription factor (*ZFP16-1* # 543, Table 2, see above) acts as a positive regulator of ADC (Vogel et al., 2005). Therefore, the low expression of *ZFP16-1* transcript in *Urea*+*NBPT* treated maize roots correlated with the downregulation of *ADC1* gene. The effect of NBPT on PAs metabolism is further supported by the inhibition of polyamine degradation (# 205, 372–373, 437, 1499, Supplementary Table 4).

A considerable modulation was observed for the catabolism of proline, an amino acid whose synthesis is metabolically linked with PA synthesis through the common precursor arginine (Balestrasse et al., 2005). Experimental evidence showed that N deficiency caused a reduction in proline levels due to the stimulation of proline catabolism (Sánchez et al., 2002; del Mar Rubio-Wilhelmi et al., 2012). Thus, the upregulation of three transcripts coding for proline oxidases (*ZmProOXs)* under the *Urea*+*NBPT* treatment (# 92–94, Table 2) might be part of a N recycling process induced within the root cells by the inhibitor.

#### Phenylalanine/tyrosine derivatives and phenylpropanoid pathway

The modulation of gene expression in response to the *Urea*+*NBPT* treatment revealed a group of transcripts involved in the shikimate pathway and the downstream reactions for the synthesis of aromatic amino acids and their derivatives in roots (Table 3, Figure 7). The aromatic amino acids, phenylalanine, and tyrosine (Rösler et al., 1997) are precursors for several secondary metabolites, including phenols (coumarin, flavonoids, lignin), spermidine derivatives/conjugates and others phenylpropanoids (Fellenberg et al., 2009; Tzin and Galili, 2010).

Previous experimental evidence had shown that under abiotic stress, such as N deficiency, the activity and content of enzymes associated with phenylpropanoid synthesis, in particular phenylalanine ammonia-lyase (PAL), increase in plants (Dixon and Paiva, 1995; Kováčik et al., 2007).

Furthermore, limited N-availability induced the expression of several genes associated with phenylpropanoid metabolisms including members of the gene families encoding enzymes such as PAL, 4-coumarate CoA ligase (4CL), and cinnamate-4-hydroxylase (C4H, Wang et al., 2003; Scheible et al., 2004; Fritz et al., 2006; Yang et al., 2011).

The addition of NBPT resulted in a downregulation of the first steps of the shikimate pathway for the synthesis of arogenate (# 193–195, Table 3) while the downstream reactions for the synthesis of cinnamic acid and phenylalanine/tyrosine-derivatives were upregulated (# 227, 232–235, 237,238, 250–251, 255–256, 258, 289, 412, Table 3, Figure 7). This behavior could be part of a N recycling mechanism. While the synthesis of arogenate requires glutamate as ammonium donor for the transamination reaction, the conversion of phenylalanine into cinnamic acid by PAL results in a release of ammonium. In this way, the activation of phenylalanine derivatives biosynthesis might help ensuring the N recycling from phenylalanine and/or tyrosine (Rösler et al., 1997), avoiding the deamination of glutamate.

Lignin synthesis was negatively modulated in *Urea*+*NBPT* treated plants (caffeate O-methyltransferase, cinnamoyl-CoA reductase and cinnamyl-alcohol dehydrogenase, # 225, 236, 252-254, Table 3, Figure 7), suggesting a metabolic shift toward the synthesis of other phenolic compounds.

As compared to *Urea* treated plants, total N and amino acids (as Gln and Asn) contents decreased in *Urea*+*NBPT* treated plants, suggesting an imbalance between C and N compounds. This cellular response is typically described under conditions of limiting N availability, where a marked shift from the N-containing molecules to carbon-rich phenols and phenylpropanoids is observed (Fritz et al., 2006; del Mar Rubio-Wilhelmi et al., 2012).

#### Modulation of genes involved in the acquisition of other nutrients

As previously reported, the urease inhibitor coordinates both Ni atoms in the urease active site (Manunza et al., 1999), thus it is plausible that in the root cells NBPT interacts with divalent cations modifying their availability for plants. Moreover, the inhibition of the urease enzyme by NBPT might also indirectly influence Ni homeostasis, with changes in the equilibrium between the active enzyme (urease binding Ni) and the apoenzyme (urease without Ni). Based on this consideration, the levels of the urease cofactor (Ni) might increase in the cytosol thus activating a response for its detoxification. Transcriptomic analyses might suggest that *Urea*+*NBPT* treated plants respond to a condition of Ni toxicity.

High concentrations of Ni are linked to a strong inhibition of the photosynthetic apparatus (chlorophyll content, thylakoid membrane, chloroplast grana structure, electron transport chain), occurring both in isolated chloroplasts and whole plant (for review see Chen et al., 2009). Besides the disruption of the photosynthetic apparatus, the toxicity symptoms due to Ni include the inhibition of CO_2_ assimilation, changes in the proline concentrations (Molas, 1997; Kozlov, 2005; Gajewska et al., 2006), and induced antioxidant enzyme and phenylalanine ammonia-lyase (PAL) activities (Yan et al., 2008). These responses might fit with the changes occurring after *Urea*+*NBPT* treatment, since the expression of *Rubisco* and other genes involved in the Calvin cycle, in proline pathway and some antioxidant and PAL enzymes were modulated by NBPT (# 58–60, 198–200, 1318–1319, 351, 466–472, 235, Supplementary Table 4, Figure 5).

In plants, metal ligands, such as nicotianamine (NA) and carboxylates (citrate and malate), act as intracellular Ni chelators (Devêvre et al., 1996; Ahonen-Jonnarth et al., 2000; Douchkov et al., 2005). The treatment with *Urea*+*NBPT* positively modulated the synthesis of the universal methyl donor *S*-adenosyl-L-methionine (SAM-S # 188, Supplementary Table 4), which along with putrescine (see above) participates to the production of NA (Herbik et al., 1999).

The availability of NA might allow maize plants to cope with the NBPT effect on metals homeostasis, favoring the transport or compartmentalization of divalent metals within the plant. In maize roots we observed a modulation of Yellow-Stripe-Like transporters (# 1335–1336, Table 4), which are homologous of Ni-NA transporters (Gendre et al., 2007).

When NBPT was added to the nutrient solution, the expression of some genes related to the homeostasis of Fe and Zn were also modulated in root cells (Table 4). Also this response might be related to changes in Ni homeostasis, a metal known to compete with Fe and Zn for NA complexation (log stability constants for complexes: Fe(II) 12.1–12.8, Zn(II) 14.6–15.4, Ni(II) 16.1, Benes et al., 1983; for review see Chen et al., 2009; Clemens et al., 2013). In particular, the root exposure to *Urea*+*NBPT* led to the upregulation of a gene coding for the vacuolar iron transporter (*ZmVIT1*, # 219; Table 4) and led to the downregulation of a putative NA transporter (*ZmZIF1*, # 2144; Table 4). The arabidopsis homologous (AtZIF1, Haydon et al., 2012) is a vacuolar membrane protein, which contributes to the intracellular distribution of NA. This protein would play an important role in Zn and Fe partitioning within the cells by limiting the sequestration of NA in the vacuole (Haydon et al., 2012). Therefore, in *Urea*+*NBPT* treated plants a limitation of metal sequestration in the vacuole might occur.

In conclusion, these results indicate that in presence of the urease inhibitor NBPT in the nutrient solution, the ureic-N assimilation is compromised and a general reprogramming of primary and secondary metabolic pathways occurs. In particular, the mechanisms involved in N-recycling appeared to be activated in treated plants (Figure 6). Moreover, root cells show a general response to compensate changes in the internal metal concentrations among different compartments. In particular changes in NA availability might led to unbalanced concentrations of Ni, Fe, and Zn.

## Author contributions

LZ and AZ performed the microarray analysis; SV and RD measured the amino acid content; LZ and NT acquired and analyzed the data. LZ, NT, ZV, RP designed and oversaw the research; LZ, NT, RP wrote the article.

## Funding

This work was supported by the Italian Ministry of University and Research (FIRB grant: RBFR127WJ9); the Department of Agricultural and Environmental Sciences, University of Udine (U.N.I.C.O. Project -2014).

### Conflict of interest statement

The authors declare that the research was conducted in the absence of any commercial or financial relationships that could be construed as a potential conflict of interest.

## Supplementary material

Supplementary Figure 1 **MapMan Pathway file**. Image file for the pathway of urea assimilation and primary metabolism without transcript bins.

Supplementary Figure 2 **MapMan Pathway file**. Image file for the pathway of phenylalanine/tyrosine derivatives without transcript bins, image adapted from Tohge et al. (2013).

Supplementary Figure 3 **Overview of up- (red) and down- (green) modulated transcripts in *Urea*+*NBPT* vs. *Urea* using *MapMan*-bincode classification**. Numbers refer to the MapMan bincode: *1*, PS; *2*, major CHO metabolism; *3*, minor CHO metabolism; *4*, glycolysis; *5*, fermentation; *6*, gluconeogenesis/glyoxylate cycle; *7*, OPP; *8*, TCA/org transformation; *9*, mitochondrial electron transport/ATP synthesis; *10*, cell wall; *11*, lipid metabolism; *12*, N-metabolism; *13*, amino acid metabolism; *14*, S-assimilation; *15*, metal handling; *16*, secondary metabolism; *17*, hormone metabolism; *18*, Co-factor and vitamine metabolism; *19*, tetrapyrrole synthesis; *20*, stress; *21*, redox; *22*, polyamine metabolism; *23*, nucleotide metabolism; *24*, Biodegradation of Xenobiotics; *25*, C1-metabolism; *26*, miscellaneous; *27*, RNA; *28*, DNA; *29*, protein; *30*, signaling; *31*, cell; *32*, micro RNA, natural antisense; *33*, development; *34*, transport; *35*, not assigned. Color scale refers to the fold change values of differentially expressed transcripts: *red* color refers to those transcripts positively regulated by *Urea*+*NBPT* treatment, while in green are transcripts negatively regulated by *Urea*+*NBPT* treatment.

Supplementary Table 1 **MapMan Mapping File**. Transcript mapping file for the pathway of urea assimilation and primary metabolism.

Supplementary Table 2 **MapMan Mapping File**. Transcript mapping file for the pathway of phenylalanine/tyrosine derivatives.

Supplementary Table 3 **Real-time RT-PCR validation of a set of genes differentially expressed in microarray analysis *Urea*+*NBPT* vs. *Urea***. In the table are shown (starting from left column): Number of transcript according to **Tables 2–4**, **Supplementary Table 4**, transcript ID, description, gene symbol, microarray value (fold change and *p*. value adj.), real-time RT-PCR values (fold change and standard deviation), sequence of forward and reverse primers and primer efficiency.

Supplementary Table4 **Differentially expressed transcripts resulted by the comparison of root transcriptional profiles of *Urea*+*NBPT* plants with root transcriptional profile of *Urea* plants (*Urea*+*NBPT* vs. *Urea* comparison)**. In the table are shown (starting from left column): transcript ID, fold change (*FC*) of *Urea*+*NBPT* vs. *Urea*, adjusted *P*-value of *Urea*+*NBPT* vs. *Urea,* gene model ID, transcript ID, protein ID, Zmays_181 ID, Zmays_181 description, homolog protein of Arabidopsis, and rice (annotation according to *Zea mays 6a* annotation file, http://genome.jgi.doe.gov), MapMan bincode and name were reported for each transcript.
