# Supplementary figures and images for: Short-Term Treatment with the Urease Inhibitor N-(n-Butyl) Thiophosphoric Triamide (NBPT) Alters Urea Assimilation and Modulates Transcriptional Profiles of Genes Involved in Primary and Secondary Metabolism in Maize Seedlings
Source: Front Plant Sci. 2016 Jun 22;7:845. doi: 10.3389/fpls.2016.00845 (PMC4916206; doi:10.3389/fpls.2016.00845)

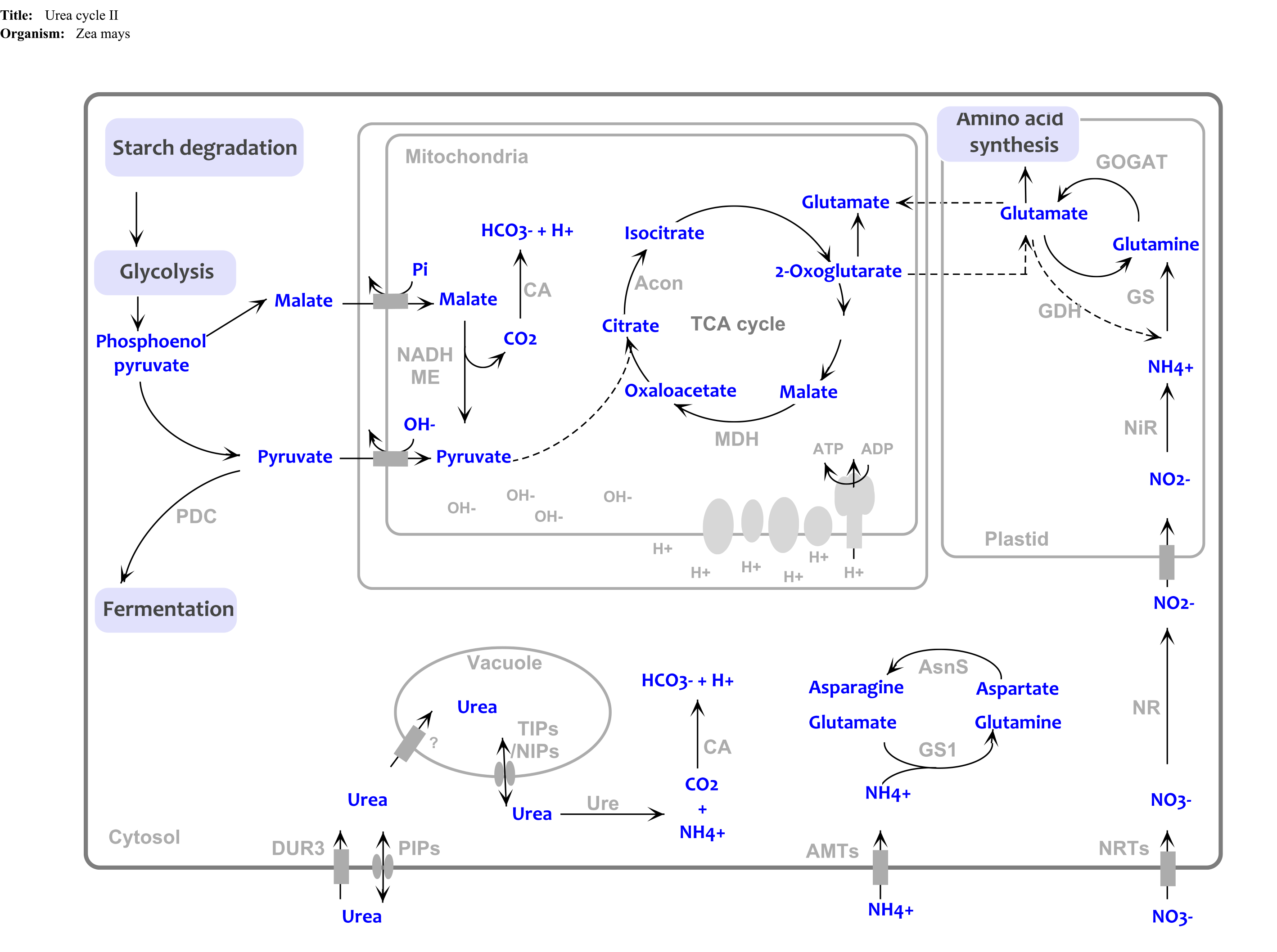

Supplement: Supplementary Figure 1 — MapMan Pathway file. Image file for the pathway of urea assimilation and primary metabolism without transcript bins. [file Image1.png]

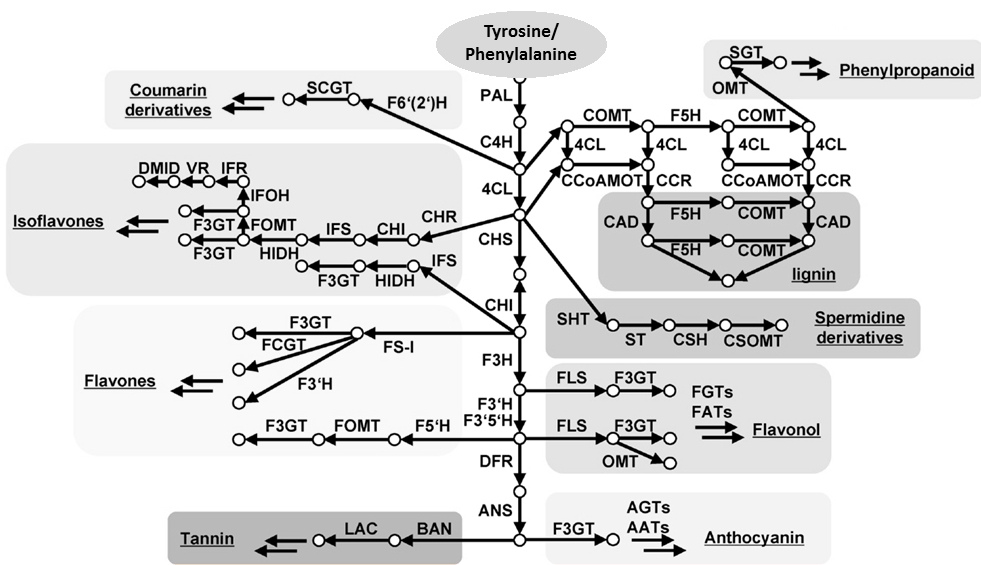

Supplement: Supplementary Figure 2 — MapMan Pathway file. Image file for the pathway of phenylalanine/tyrosine derivatives without transcript bins, image adapted from Tohge et al. (2013). [file Image2.JPEG]

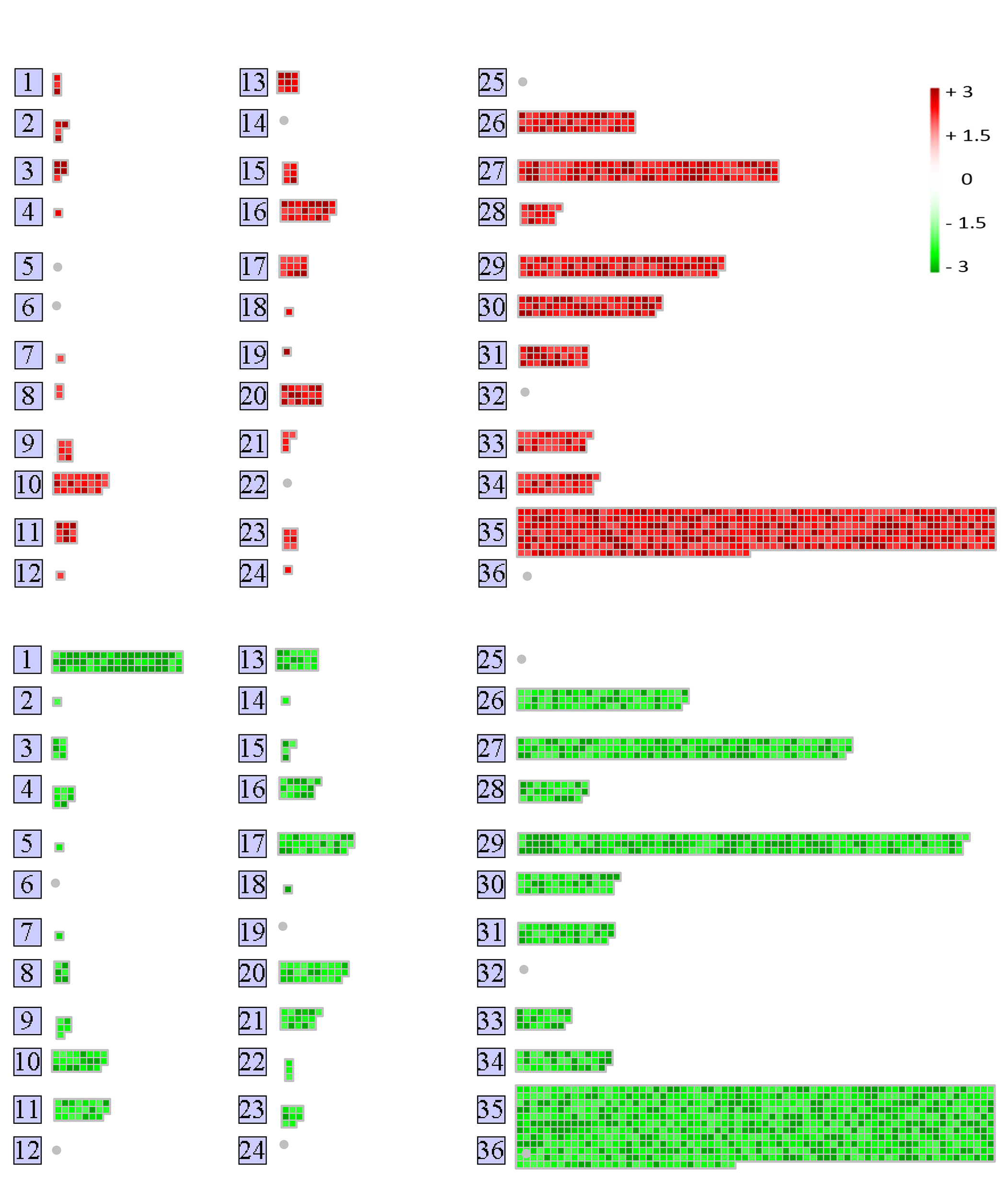

Supplement: Supplementary Figure 3 — Overview of up- (red) and down- (green) modulated transcripts in Urea+NBPT vs. Urea using MapMan-bincode classification. Numbers refer to the MapMan bincode: 1, PS; 2, major CHO metabolism; 3, minor CHO metabolism; 4, glycolysis; 5, fermentation; 6, gluconeogenesis/glyoxylate cycle; 7, OPP; 8, TCA/org transformation; 9, mitochondrial electron transport/ATP synthesis; 10, cell wall; 11, lipid metabolism; 12, N-metabolism; 13, amino acid metabolism; 14, S-assimilation; 15, metal handling; 16, secondary metabolism; 17, hormone metabolism; 18, Co-factor and vitamine metabolism; 19, tetrapyrrole synthesis; 20, stress; 21, redox; 22, polyamine metabolism; 23, nucleotide metabolism; 24, Biodegradation of Xenobiotics; 25, C1-metabolism; 26, miscellaneous; 27, RNA; 28, DNA; 29, protein; 30, signaling; 31, cell; 32, micro RNA, natural antisense; 33, development; 34, transport; 35, not assigned. Color scale refers to the fold change values of differentially expressed transcripts: red color refers to those transcripts positively regulated by Urea+NBPT treatment, while in green are transcripts negatively regulated by Urea+NBPT treatment. [file Image3.TIF]
